# Hospice palliative care utilization among patients with cancer and non-cancer lung diseases: a retrospective cohort study

**DOI:** 10.1186/s12904-026-02256-0

**Published:** 2026-07-31

**Authors:** Zi-Jun Chen, Ru-Yih Chen, Chun-Hao Yin, Ying-Chun Li, Min-Hsi Lin

**Affiliations:** 1 Department of Family Medicine, Kaohsiung Veterans General Hospital, Kaohsiung, Taiwan, ROC; 2 Department of Pharmacy, Chia Nan University of Pharmacy & Science, Tainan, Taiwan, ROC; 3 Department of Business Management, Institute of Health Care Management, National Sun Yat-Sen University, Kaohsiung, Taiwan, ROC; 4 Department of Medical Education and Research, Kaohsiung Veterans General Hospital, Kaohsiung, Taiwan, ROC; 5 Division of Chest Medicine, Kaohsiung Veterans General Hospital, Kaohsiung, Taiwan, ROC

**Keywords:** Lung disease, Cancer, Non-cancer disease, Hospice palliative care, Predictive model, End-of-life care

## Abstract

**Background:**

Disparities in hospice palliative care utilization rates between terminally ill cancer and non-cancer patients are evident. However, limited research has examined factors associated with hospice palliative care utilization among patients with cancer and non-cancer lung diseases. We aimed to compare hospice palliative care utilization between patients with cancer and those with non-cancer lung diseases and to identify factors associated with hospice palliative care utilization.

**Methods:**

We conducted a retrospective cohort study of deceased patients with primary lung disease diagnoses at a southern Taiwanese tertiary teaching hospital between January 2019 and December 2020. Demographic and clinical data were extracted from the hospital’s medical information system. Multivariate logistic regression analyses were performed to identify factors associated with hospice palliative care utilization.

**Results:**

The study included 464 non-cancer and 269 cancer patients, averaging 77.4 ± 13.4 years. While there was no significant difference in the rate of the Do-Not-Resuscitate code (95.08% vs. 91.81%, *p* = 0.065), the hospice palliative care rate differed significantly (36.89% vs. 55.97%, *p* < 0.001) between the two groups. Multiple logistic regression analysis revealed increased odds of hospice palliative care utilization associated with cancer diagnosis, higher education level, urban residency, and autonomous Do-Not-Resuscitate decisions. Decreased odds were associated with being male, emergency hospital admission, and Charlson comorbidity index scores ≥ 2. Based on the logistic regression findings, we create a nomogram that serves as a predictive tool for assessing patients’ utilization of hospice palliative care services.

**Conclusion:**

By analyzing medical records, this study highlights a disparity in utilization between cancer and non-cancer patients. The development of a nomogram prediction model can enable the identification of patients whose hospice palliative care needs might have been inadvertently overlooked at admission. We hope to encourage medical practitioners to be more aware of patients’ needs early to ease physical and emotional distress.

## Background

Patients with advanced non-cancer lung disease face symptoms comparable to those encountered by individuals with advanced cancer. These symptoms encompass a range of challenges, including difficulty breathing, fatigue, weakness, unintentional weight loss, and chest discomfort [1]. Alongside these physical burdens, patients also grapple with psycho-spiritual complexities such as anxiety, fear, depression, and a loss of hope. Moreover, feelings of social isolation, loneliness, and introspection about life’s purpose are common [2]. These patients require hospice palliative care (HPC) as intricate and comprehensive as that provided to cancer patients [3, 4]. Existing evidence demonstrates the efficacy of HPC services in enhancing the quality of life for both patient groups [5–9].

HPC is pivotal in healthcare by significantly enhancing the quality of life for patients and their families facing life-threatening illnesses. Addressing physical, psychological, social, and spiritual well-being provides comprehensive support during challenging times. With the global aging population and the increasing burden of both communicable and non-communicable diseases, the demand for hospice palliative care is projected to rise, underscoring its growing significance within healthcare systems [10, 11].

However, patients with non-cancer terminal illnesses often receive HPC later in the disease trajectory and at a lower rate than cancer patients for various reasons [12–17]. These reasons encompass misconceptions and limited awareness about HPC, often perceived as exclusive to cancer patients. This creates challenges in determining the appropriate timing to initiate HPC for non-cancer critical conditions characterized by unpredictable disease trajectories and uncertain prognoses [18]. Additionally, the availability and integration of HPC services can vary across healthcare settings and regions, resulting in restricted access to dedicated resources for non-cancer patients.

Patients with both terminal lung cancer and non-cancer-related illnesses can benefit from dedicated care and support provided by the same medical team within their unit. Nevertheless, not all patients are equally referred to HPC before reaching the end of life [19]. Recognizing the significance of equitable access to palliative care for all patients, addressing these discrepancies becomes imperative to ensure patient-centric care and foster collaborative decision-making. This research examines the variables influencing HPC referrals. It proposes strategies to optimize the referral process, thereby mitigating the disparities in the utilization of hospice palliative care among individuals afflicted with terminal lung diseases.

## Methods

### Data sources

Using the hospital information system of a public medical center in Taiwan, we identified patients who were admitted and later deceased due to lung disease between January 1, 2019, and December 31, 2020. We collected comprehensive information about their care during the twelve months preceding their passing, including demographic details and electronic medical records (EMR). These records encompassed scanned documents related to “Do-Not-Resuscitate” (DNR) decisions and records of Hospice Palliative Care (HPC) referrals.

Patients diagnosed with lung cancer were identified through their discharge records, which featured the primary disease classification ICD-10 code C34. In contrast, patients with non-cancer lung diseases were identified using ICD-10 codes J00-C99. In Taiwan, in addition to patients who die in hospital, some terminally ill patients are discharged shortly before death in accordance with cultural practices and family preferences. Consequently, patients with an impending death discharge were alive at the time of hospital discharge but died shortly thereafter. Therefore, discharge status was classified as either “expired” or “impending death discharge”, with both categories representing patients who were deceased for the purposes of this study.

Based on the additional diagnosis codes present in the EMR, we calculated the Charlson Comorbidity Index Score (CCIS). This score allowed us to assess the severity and impact of patients’ comorbidities [20].

### Identification of terminal disease and hospice palliative care status

In Taiwan, cardiopulmonary resuscitation is obligatory for all dying patients unless two physicians have verified their terminal illness and a DNR order is issued. Before this order is given, the physician must obtain a patient-signed DNR letter of intent. If a terminally ill patient loses consciousness or cannot communicate their wishes, a close relative can sign a consent form on their behalf. Physicians who violate this regulation may face penalties, practice suspension, or even license revocation [21]. The DNR document confirms the terminal diagnosis and determines whether the DNR decision was made autonomously. Therefore, our research utilizes the DNR code to identify terminally ill patients and examines DNR documents to assess the autonomy of the decision.

According to Taiwan’s *Hospice Palliative Care Act*, HPC refers to palliative and supportive medical care provided to alleviate the physical, psychological, and spiritual suffering of terminally ill patients and to improve their quality of life [21]. Although the terms palliative care and hospice care are closely related concepts, they are not entirely synonymous. Palliative care aims to improve quality of life and relieve suffering throughout the course of a serious illness, whereas hospice care focuses on comfort-oriented care for patients approaching the end of life [22]. Taiwan’s National Health Insurance provides coverage for HPC services, including homecare, shared-care consultation, and hospice ward admission. In this study, patients assigned these claim codes within a year before their death are classified as HPC recipients in the database, regardless of where they received the care.

### Statistical analysis

Demographic and clinical characteristics of the entire analytical population were summarized and categorized into two groups: patients with DNR orders receiving HPC and patients with DNR orders without HPC. Differences between these groups were evaluated using independent samples t-test for continuous variables and χ2 tests for categorical variables. Subsequently, logistic regression analysis was performed, treating patients with DNR orders and their HPC status as binary dependent variables. A purposeful selection process was employed, which began with a univariate analysis of the collected parameters. Variables that exhibited significance at a p-value < 0.10 were selected as candidates for the multivariate analysis [23]. Stepwise logistic regression was then utilized to assess the influence of various factors, and the results were presented using a forest plot.

Furthermore, a nomogram plot was constructed using the stepwise logistic regression model. This nomogram estimates the probability of HPC according to identified significant predictors. The model’s predictive performance was evaluated using the maximum area under the receiver operating characteristic (ROC) curve. All statistical analyses were conducted using SAS version 9.4 and STATA 14. A p-value of < 0.05 was considered statistically significant.

## Results

### Patient characteristics

A total of 783 lung disease patients who received treatment at this medical center passed away between January 1, 2019, and December 31, 2020. Comparative analysis of demographic and clinical data revealed a statistically significant age difference between the non-cancer and cancer groups (average age of 79.34 years vs. 73.96 years, *p* < 0.001). While there was no statistically significant difference in DNR code prevalence (95.08% vs. 91.81%, *p* = 0.065), notable variations were observed in the coverage rate of HPC, the frequency of last admissions through the emergency room, and the CCIS. Detailed information can be found in Table 1.

**Table 1:** Demographic and clinical characteristics comparison between deceased cancer and non-cancer patients with lung diseases (*N* = 783)

Variables	Category/ Mean ± SD	Non-cancer	Cancer	*p*
Demographic characteristics				
Age (years)	77.36 ± 13.39	79.34 ± 0.59	73.96 ± 0.85	< 0.001
Age group (years)	< 65	78 (15.98)	73(24.91)	0.002
	≥ 65	410 (84.02)	220 (75.09)	
Gender	Female	132 (27.05)	96 (29.35)	0.487
	Male	356 (72.95)	207 (70.65)	
Marriage	Yes	203 (41.06)	130 (44.37)	0.448
	Other	285 (58.40)	163 (55.63)	
Children	No	47 (9.63)	27 (9.22)	0.848
	Yes	441 (90.37)	266 (90.78)	
Residency	Urban	44 (9.02)	29 (9.90)	0.682
	Rural	444 (90.98)	264 (90.10)	
Clinical characteristics				
Do-Not-Resuscitate code	No	24 (04.92)	24 (08.19)	0.065
	Yes	464 (95.08)	269 (91.81)	
Hospice Palliative care	No	308 (63.11)	129 (44.03)	< 0.001
	Yes	180 (36.89)	164 (55.97)	
Emergency hospital admission	No	58 (11.89)	238 (18.77)	0.008
	Yes	430 (88.11)	28 (81.23)	
Ward	General	337 (69.06)	161 (54.95)	< 0.001
	ICU	100 (20.49)	47 (16.04)	
	Hospice	51 (10.45)	85 (29.01)	
Discharge status	Expired	411 (84.22)	20 (81.91)	0.401
	Impending death	77 (15.78)	53 (18.09)	
Length of stay (days)	14.66 ± 14.22	14.28 ± 0.67	14.65 ± 0.75	0.725
CCIS	1.81 ± 2.09	1.98 ± 0.10	1.47 ± 0.10	0.001

*SD *standard deviation, *ICU *intensive care unit, *CCIS *Charlson comorbidity index score

**Fig. 1:**
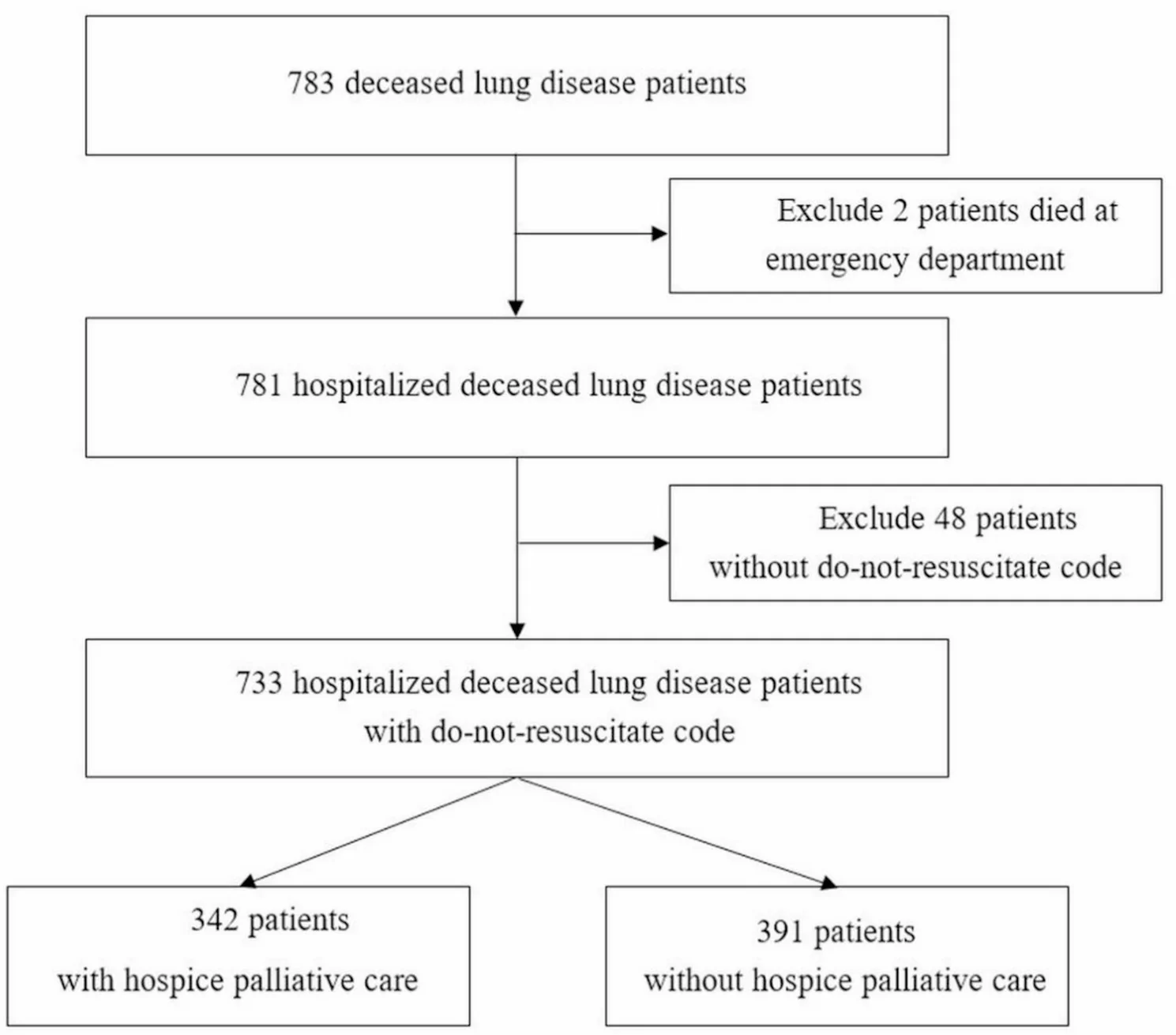
Flow chart of the patient selection process

Among these patients, 733 individuals with DNR codes were included in our study. The flowchart in Fig. 1 illustrates the subject inclusion and exclusion process. The average age of these 733 patients was 77.4 ± 13.4 years. Among them, 464 were diagnosed with non-cancer diseases, while 269 were diagnosed with cancer. This distribution aligns with statistics reported in the literature, where the number of non-cancer patients exceeds that of cancer patients.

### Factors influencing hospice palliative care utilization and nomogram

The cohort of 733 patients was classified into two groups based on their receipt of HPC. Significant disparities were observed in various factors, including age, gender, residency, education level, CCIS, cancer diagnosis, autonomous DNR decision, and admission through the emergency department. For comprehensive details, please refer to Table 2. Univariate logistic regression was performed to identify potential influencing factors for HPC, as outlined in Table 3.

**Table 2:** Comparison analysis between DNR patients with and without hospice palliative care (*N* = 733)

Variables	Total	With (*N* = 342)	Without (*N* = 391)	*p*
	Mean ± SD/ *N* (%)	Mean ± SD/ *N* (%)	Mean ± SD/ *N* (%)	
Age (years)	77.4 ± 13.4	74.5 ± 14.0	79.9 ± 12.3	< 0.001
Length of stay (days)	14.7 ± 14.2	18.8 ± 16.0	11.0 ± 11.3	< 0.001
First DNR to death (days)	84.3 ± 226.3	80.1 ± 213.0	87.9 ± 237.5	0.639
Male	525 (72)	234 (68)	291 (74)	0.072
Marriage	422 (58)	199 (58)	223 (57)	0.753
With children	668 (91)	311 (91)	357 (91)	0.861
Veterans	246 (34)	103 (30)	143 (37)	0.065
Urban resident	670 (91)	323 (94)	347 (89)	0.006
Education				< 0.001
No formal education	151 (21)	52 (15)	99 (25)	
Primary to high school	481 (66)	226 (66)	255 (65)	
Diploma or above	101 (14)	64 (19)	37 (10)	
Age ≥ 65 years				< 0.001
No	140 (19)	87 (25)	53 (14)	
Yes	593 (81)	255 (75)	338 (86)	
CCIS	1.8 ± 2.1	1.5 ± 1.9	2.1 ± 2.2	< 0.001
Cancer	269 (37)	163 (48)	106 (27)	< 0.001
Autonomous DNR decision	221 (30)	158 (46)	63 (16)	< 0.001
Emergency hospital admission	626 (85)	264 (77)	362 (93)	< 0.001

*SD *standard deviation, *DNR *Do-Not-Resuscitate, *CCIS *Charlson comorbidity index score

**Table 3:** Univariate logistic analysis of factors influencing hospice palliative care

Variables	OR (95% CI)	*p*
Age ≥ 65 years	0.46 (0.32–0.67)	< 0.001
Male	0.75 (0.54–1.03)	0.072
Marriage	1.05 (0.78–1.41)	0.753
Children	0.96 (0.57–1.59)	0.861
Veterans	0.75 (0.55–1.02)	0.065
Urban residency	2.16 (1.23–3.77)	0.007
Education		
No formal education	reference	
Primary to high school	1.69 (1.15–2.47)	0.007
Diploma or higher	3.29(1.95–5.57)	< 0.001
CCIS ≥ 2	0.51 (0.38–0.69)	< 0.001
Cancer	2.45 (1.80–3.33)	< 0.001
Autonomous DNR decision	4.47 (3.17–6.30)	< 0.001
Emergency hospital admission	0.27 (0.17–0.43)	< 0.001

*OR *odds ratio, *CI *confidence interval, *DNR *Do-Not-Resuscitate, *CCIS *Charlson comorbidity index score

**Fig. 2:**
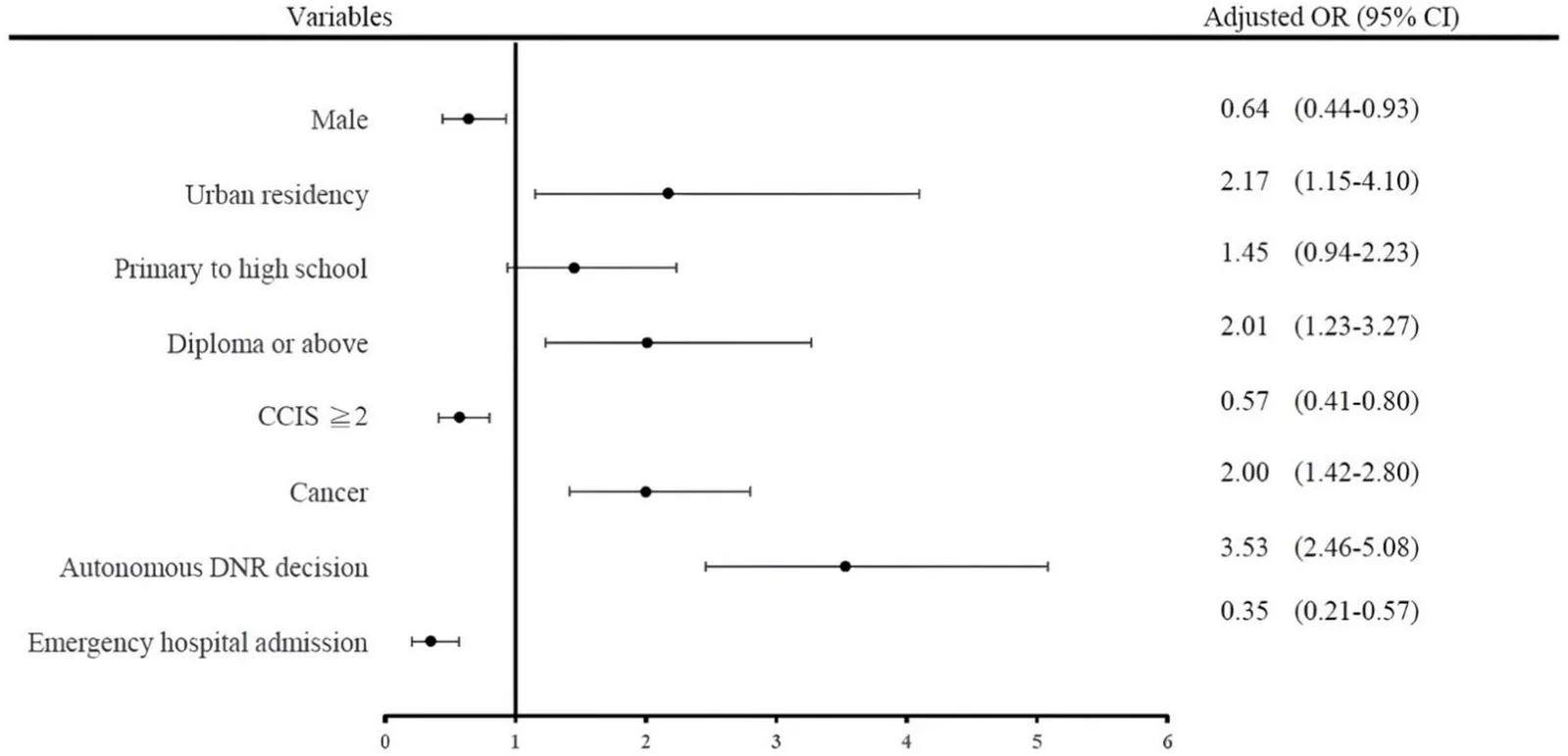
Forest plot of stepwise logistic regression of factors influencing hospice palliative care. OR: odds ratio; CI: confidence interval; CCIS: Charlson comorbidity index score; DNR: Do-Not-Resuscitate

Further stepwise logistic regression unveiled the following as significant factors: male gender (odds ratio [OR] 0.64, 95% confidence interval [CI] 0.44–0.93), urban residency (OR 2.17, CI 1.15–4.10), college education (OR 2.01, CI 1.23–3.27), CCIS ≥ 2 points (OR 0.57, CI 0.41–0.80), cancer diagnosis (OR 2.00, CI 1.42–2.80), autonomous DNR decision (OR 3.53, CI 2.46–5.08), and emergency hospital admission (OR 0.35, CI 0.21–0.57). The average variance inflation factor value was 1.15, indicating minimal multicollinearity concerns. The p-value obtained from the Hosmer–Lemeshow test was 0.469 (*X*^2^ = 6.00), signifying a well-fitting model. Detailed results of the multivariate regression are displayed in the forest plot shown in Fig. 2.

**Fig. 3:**
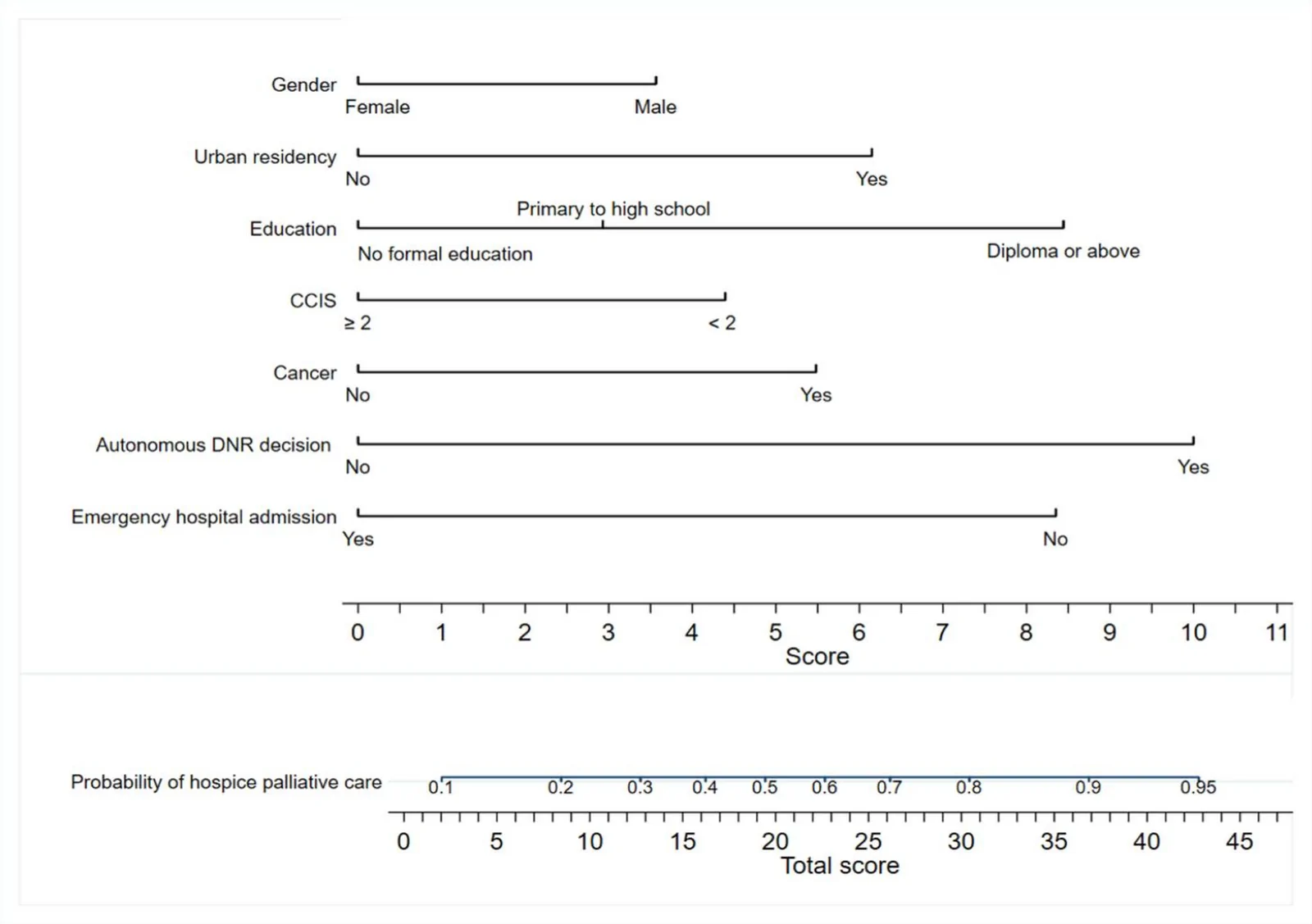
Nomogram plot for predicting the probability of hospice palliative care for terminal lung disease patients. For each patient, there’s a corresponding point on the 8th row (score) for each variable. The total score is calculated by adding all the facts, as shown in the bottom row (9th row). By drawing a vertical line from the total score to the 9th row, the probability of hospice palliative care for that patient can be determined. CCIS: Charlson comorbidity index score; DNR: Do-Not-Resuscitate

Utilizing the final logistic regression outcomes, a predictive nomogram was developed (Fig. 3) to assess the likelihood of HPC for individual patients. This nomogram incorporates seven factors assigned varying points (0, 1, or 2) based on patient demographic and clinical data. By utilizing this nomogram, healthcare professionals can estimate the relative probability of a patient’s referral to HPC. The nomogram demonstrates robust predictive capacity, with an area under the ROC curve value of 0.784 (95% confidence interval: 0.752–0.817), indicating a moderate level of predictive accuracy.

## Discussion

The study delved into HPC utilization among patients with lung disease and investigated the influencing factors. Although there were no significant differences in DNR codes between cancer and non-cancer groups, the hospice palliative care utilization rate was significantly higher among terminal cancer patients. Stepwise logistic regression revealed several key factors affecting HPC utilization, including gender, residency, education, CCIS, cancer diagnosis, autonomous DNR decisions, and emergency admissions. A predictive nomogram was created based on these factors to estimate the likelihood of HPC referral.

### Positive influencing factors

#### Cancer diagnosis

As demonstrated in prior studies, the prevalence of HPC referrals has historically been higher among patients diagnosed with cancer than those with non-cancer conditions [13–15]. This study further substantiates this trend, revealing that patients with terminal cancer diagnoses exhibited a 2 times higher odds ratio of receiving HPC than those with non-cancer lung diagnoses. This finding underscores the pronounced disparities in accessing HPC between patients facing terminal lung cancer and those without cancer diagnoses.

Patients diagnosed with terminal lung cancer often follow a predictable health decline trajectory, with a life expectancy within 6–12 months. HPC offers tailored, comprehensive support that addresses the unique needs of cancer patients during this phase. Given the historical emphasis on cancer-centric HPC, healthcare providers are more attuned to referring cancer patients for such care, thus creating a discernible knowledge gap concerning the provision of HPC for non-cancer terminal illnesses. This gap can influence referral patterns, with non-cancer diseases receiving relatively less societal recognition of HPC’s importance than cancer. Consequently, attention tends to be confined to ailment-specific treatments for terminally ill non-cancer patients, while the fundamental requisites of HPC may receive comparatively reduced focus [24].

Patients grappling with non-cancer terminal diseases often navigate a more erratic and unpredictable disease trajectory. This characteristic challenges healthcare professionals in determining the optimal timing to initiate end-of-life care conversations [25]. The conventional focus of medical practitioners on interventions aimed at prolonging life can hinder proactive discussions about HPC initiation. As a result, patients and their families might remain unaware of the viable option of HPC, even when encountering distressing symptoms similar to those experienced by patients with terminal cancer. Consequently, patients with terminal lung cancer are more predisposed to receiving HPC than their non-cancer counterparts.

Equal access to HPC requires awareness campaigns for healthcare professionals and the public, specialized training, and a broader scope beyond cancer. This is vital for fair access to care and better end-of-life experience.

#### Education level and urban residency

Individuals with a diploma or higher education level exhibit a greater propensity to comprehend that HPC doesn’t signify a withdrawal from medical treatment but instead embraces a medical approach focused on enhancing the quality of life. This understanding leads to heightened acceptance and active pursuit of HPC services. Similarly, residents of urban areas typically enjoy improved access to medical information and are more inclined to acknowledge the limits of medical interventions. Therefore, they are often more receptive to recalibrating treatment objectives and considering the merits of HPC. These findings align with earlier research, further corroborating their significance [24, 26–31].

Many individuals, particularly those with limited education, living in rural areas, or from specific cultures, may not comprehensively understand HPC. This can result in underutilization of HPC services, depriving those needing additional medical resources to alleviate their suffering during end-of-life. To ensure equal access to appropriate care, public awareness campaigns and educational initiatives should empower rural residents with comprehensive knowledge of HPC services.

#### Autonomous DNR decision

Autonomous DNR decisions signify that patients possess a comprehensive grasp of their medical limitations and have willingly embraced them. This fosters improved communication with the medical staff and provides patients with a platform to articulate their physical and psycho-spiritual requirements. Subsequently, the medical team can engage in meaningful discussions with patients regarding referrals, amplifying the potential for HPC utilization.

Engaging patients in conversations about autonomous DNR decisions holds particular significance when there is a potential for life-threatening events to occur within one month [32]. Effective communication between patients and healthcare providers facilitates shared understanding, comprehensive care planning, and decision-making that aligns with patients’ preferences and values, empowering patients to actively shape their care journey and align medical interventions with their end-of-life goals and aspirations.

### Negative influencing factors

#### Male gender

The study’s findings underscore a significant gender-based disparity in the utilization of HPC, with males being less likely to access these services than females. This aligns with a meta-analysis highlighting barriers to HPC services among cancer patients [24]. The reasons underlying this trend are multifaceted and extend beyond mere medical considerations.

Male underutilization of HPC services is partly attributed to societal expectations of self-reliance and discomfort with discussing health concerns. Additionally, a preference for aggressive anti-disease treatments may lead men to overlook HPC options that could improve their quality of life during their final phase.

To address gender disparity in HPC services, we need targeted strategies for males. Educate males on their concerns, involve community leaders, and provide support groups. Open and transparent communication is also necessary to dispel HPC myths and reduce stigma.

#### Emergency hospital admission

The emergency department’s end-of-life care lacks a standardized screening process and timely support [17, 33]. Strategies to improve the quality of care can include staff champions and education for medical staff to provide first-aid symptom control and consult the HPC team promptly [34–36]. However, the lack of evidence regarding the implementation and use of these strategies in clinical environments and their effects impedes the assessment of their overall impact.

Patients admitted through the emergency department may not understand the severity of their illness or be prepared for a sudden decline in their condition. To improve this, early discussions about healthcare options should be initiated before emergencies occur, especially in cases with poor prognosis. The Supportive and Palliative Care Indicator Tool (SPICT) is designed to help healthcare professionals recognize patients with deteriorating health who might have HPC needs [37]. Using SPICT for screening could be a practical approach to identify the targeting patients in routine care.

#### Comorbidities

The association between patients with multiple comorbidities and their preparedness for end-of-life care might seem intuitive, as their heightened risk of fatal outcomes could suggest a greater understanding of HPC. However, our study findings, consistent with previous studies, have revealed a paradoxical trend where patients with higher CCIS are less likely to receive HPC services [38–42].

Research indicates that individuals burdened with multiple chronic illnesses often lack the necessary preparedness for end-of-life care. This could be attributed to an insufficient understanding of health information relevant to this stage of life. Additionally, individuals with multiple comorbidities may have their DNR choices determined by their family members due to their diminished mental capacity. Those needing HPC may struggle to express their physical and psycho-spiritual distress, leading to neglect from medical staff [43].

Patients enrolled in our study had DNR codes, indicating their awareness of worst-case scenarios. Some may choose to forgo further treatment and let nature take its course. They may consider HPC could result in unwanted life-prolonging potentially. Therefore, despite the potential benefits of HPC, some patients may choose not to pursue it. Such thoughts could make them miss the chance to have HPC to manage their respiratory symptoms and other physical distress, anxiety, and depression and provide emotional and spiritual support regarding patients and their loved ones’ needs, wishes, and beliefs, including grief counseling.

### Nomogram for predicting hospice palliative care utilization for patients with end-stage lung disease

A nomogram is a graphical representation of multivariate models for prognostic prediction [44]. Based on our stepwise logistic regression findings, we have crafted a nomogram in Fig. 3 that serves as a predictive tool for assessing patients’ utilization of HPC services.

Consider a practical scenario: a 72-year-old female patient with a primary level of education residing in an urban area diagnosed with mild dementia and diabetic nephropathy. Her hospital admission was via the emergency department due to severe pneumonia, and her condition deteriorated, leading to imminent respiratory failure. The patient’s family decided on a DNR course of action. Upon evaluating the patient’s characteristics, her total score on the nomogram approximated 10 points. Consequently, the calculated probability of her seeking hospice palliative care was under 20%. In such instances, it is advisable to implement a medical reminder system to promptly alert the medical team about the patient’s HPC needs.

By leveraging this tool to enhance decision-making and facilitate early interventions, healthcare providers can ensure that individuals with end-stage lung disease receive the appropriate care and support that aligns with their preferences and needs.

### Strengths and limitations

Our study introduces a novel approach to identifying overlooked cases of HPC utilization for terminal lung diseases, including both cancer and non-cancer patients, through a nomogram scoring method that considers various factors. However, there are limitations. Firstly, using the ICD code to determine the primary cause of death for patients with multiple comorbidities could introduce some selection bias. However, disease classification relied on the physician’s death certificate, a reliable source, and certified personnel. Secondly, the study database was limited to a single hospital, requiring further research for generalizability. Third, symptom-related variables were unavailable in our study. The clinical value of the nomogram lies in identifying patients with a DNR preference who are less likely to receive HPC and may therefore be overlooked for evaluation of HPC needs. The model serves as a screening tool to prompt further assessment of symptom burden and psychosocial needs. Future research should integrate standardized symptom assessments to better evaluate the appropriateness and impact of HPC referral.

## Conclusion

When clinicians focus exclusively on organ-specific treatments, the holistic care needs of patients are often neglected. Our study endeavors to pinpoint the factors that impact the utilization of HPC among individuals afflicted with terminal lung disease. It reveals the discrepancy in HPC usage and offers a nomogram prediction model. We hope our study results will heighten medical professionals’ awareness regarding terminally ill patients’ HPC needs while hospitalized to deliver superior patient centered care promptly.

## Data Availability

The datasets used and/or analyzed during the current study are available from the corresponding author on reasonable request.

## Abbreviations

CCIS: Charlson Comorbidity Index Score
CI: Confidence interval
DNR: Do-Not-Resuscitate
EMR: Electronic medical records
HPC: Hospice Palliative Care
OR: Odds ratio
